# Comparison of antemortem clinical diagnosis and post-mortem findings in intensive care unit patients

**DOI:** 10.1007/s00428-020-03016-y

**Published:** 2021-02-13

**Authors:** Stefan Rusu, Philomène Lavis, Vilma Domingues Salgado, Marie-Paule Van Craynest, Jacques Creteur, Isabelle Salmon, Alexandre Brasseur, Myriam Remmelink

**Affiliations:** 1 Hôpital Erasme, Department of Pathology, Université Libre de Bruxelles, Brussels, Belgium; 2 Hôpital Erasme, Department of Intensive Care, Université Libre de Bruxelles, Brussels, Belgium; 3 Centre Universitaire Inter Regional d’Expertise en Anatomie Pathologique Hospitalière (CurePath), Charleroi (Jumet), Belgium; 4 DIAPath - Center for Microscopy and Molecular Imaging, Université Libre de Bruxelles, Gosselies, Belgium

**Keywords:** Autopsy, Intensive care, Diagnostic discrepancy, Post-mortem examination, Goldman

## Abstract

Autopsy is an important quality assurance indicator and a tool to advance medical knowledge. This study aims to compare the premortem clinical and postmortem pathology findings in patients who died in the Intensive Care Unit (ICU), to analyze if there are any discrepancies between them, and to compare the results to two similar studies performed in our institution in 2004 and 2007. Between January 1, 2016, and December 31, 2018, 888 patients died in the ICU and 473 underwent post-mortem examination (PME) of whom 437 were included in the present study. Autopsies revealed discrepancies between clinical diagnosis and pathologic findings according to in 101 cases (23.1%) according to Goldman classification. Forty-eight major discrepancies (class I and class II) were identified in 44 cases and the most frequent identified discrepancies were pulmonary embolism (3/12) as class I and malignancies (13/35) as class II. They were more frequent in patients hospitalized for less than 10 days then in the group with more than 10 days of hospitalization (13.8% vs 4.5%; *p =* 0.002). No statistical difference has been noticed concerning age, gender, and ICU stay. We observed an increase of performed autopsies and a total discrepancy rate similar to the studies performed in the same institution in 2004 (22.5%) and 2007 (21%). In conclusion, discrepancies between clinical and PME diagnoses persist despite the medical progress. Secondly, the autopsy after a short hospital stay may reveal unexpected findings whose diagnosis is challenging even if it may be suspected by the intensivist.

**Supplementary Information:**

The online version contains supplementary material available at 10.1007/s00428-020-03016-y.

## Introduction

Autopsy is considered an important tool to evaluate the presence and extent of disease, to advance medical knowledge, to improve clinical practice, to train young physicians, especially for pathology trainees, but not only to teach students the fundamentals of anatomy and pathology. Moreover, it is an important quality assurance indicator of patient management, in terms of diagnosis and treatment [1, 2]. There is a considerable amount of studies comparing the accuracy of clinical diagnosis over the years and despite all the progress and new diagnostic resources available, errors in diagnosis still occur [3–6]. The persistence of discordances between clinical and post-mortem diagnosis (PME) advocates the continuous autopsy practice [7–9]. Moreover, studies carried out in the last three decades in different groups of patients (neonatal, pediatric, psychiatric, geriatric patients) in university hospitals or not, failed to show a meaningful increase between antemortem and post-mortem diagnosis [1, 7, 9–17].

Although it is a powerful tool to improve medical practice quality, hospital autopsy rates declined worldwide over the last 30 years. It decreased from 37.3% in the 1990s to 27.75% in 2018 in the World Health Organization (WHO) European region according to WHO-European Health Information Gateway [18]. The rate halved in the European Union’s countries (from 23.8% in 1989 to 10% in 2018). In Belgium, this downward trend is even more significant, the hospital autopsy rate reduced almost seven times in the last 20 years (from 18.9% to 2.2%) [18]. The decreased rate could be explained partly by the modern diagnostic techniques, especially imaging, allowing rapid and efficient diagnosis. On the other side, reluctance to ask relatives for the consent, fear of medico-legal implications, reluctance of the pathologists because of the possible infectious risk, and delays in the communication of autopsy results also contribute to the decreasing trend of performing autopsies [7, 15, 19–21]. Furthermore, economic reasons may depreciate the need for autopsies [13, 22, 23]. Previous assessment of agreement rates between antemortem and post-mortem diagnosis in our university hospital in Belgium was made in 2004 [24] and 2007 [7].

### Aim of the study

The main purpose of this study is to compare the premortem clinical diagnoses and postmortem pathology findings in patients who died in the Intensive Care Unit (ICU) and to analyze if there are discrepancies between them. The obtained results are compared to two similar studies performed in our institution and published in 2004 and 2007, respectively.

### Patients and methods

Clinical and post-mortem findings of all patients who died in the ICU of Erasme University Hospital, Belgium, from January 1, 2016, to December 31, 2018, and underwent PME were retrieved and reviewed. Erasme Hospital is a tertiary referral hospital with 1048 beds and 25,000-30,000 patients hospitalized each year. The Intensive Care Department includes five medico-surgical ICUs and a four-bed shock lab (designed for critical patients’ stabilization). The reported mortality rate is around 12%. Full-body autopsies were performed within 48 h of death (frequently within 24 h). The procedure included macroscopic and microscopic assessment of all internal organs and brain if indicated in the autopsy request. PMEs are performed following an internal standardized procedure (the reference book is *An introduction to autopsy technique, 2*^*nd*^ *edition*) [25]*.* Data retrieved from medical charts included age, sex, length of hospitalization (ICU and/or hospital stay), and the major clinical findings (which include the immediate cause of death, the underlying cause of death, and contributory causes). The autopsy report included all histological and immunohistochemical findings. Doubtful cases were reviewed by a second pathologist in order to avoid subjectivity and misinterpretation of the histological findings. The exclusion criteria were age < 18, incomplete/partial autopsy, or refusal to autopsy as a request by the referral clinician, relatives, and donor body autopsies.

The comparison between antemortem clinical diagnoses and postmortem histological findings for each patient was realized by an ICU doctor. When discrepancies were identified, they were sorted into five classes according to the classification proposed by Lee Goldman et al. [8] (Table 1). When multiple unexpected findings were retrieved, only the most severe level of discrepancy was considered, and when multiple unexpected findings presented the same level of discrepancy all unexpected findings were considered for statistical analysis. The present study used the same methodology as the one performed previously in our institution by Maris et al. [7].

**Table 1:** Goldman classification regarding the discrepancies between clinical and histopathological findings in autopsies

*Class*	*Discrepancy degree*	*Explanation*
I	Major	A missed diagnosis (not suspected or because the tests were inconclusive, misleading, not available, or misinterpreted) that would have changed the patient management leading to a cure or prolonged survival
II	Major	A missed diagnosis of which detection before death would not have probably led to changes in ingoing patient care
III	Minor	A missed diagnosis linked to the terminal disease process but not directly associated with the cause of death
IV	Minor	A missed unrelated diagnosis that might eventually have affected prognosis
V	Complete agreement	

The cause of death was evaluated in each case and included in one of the following 12 categories: sepsis/peritonitis, cardiopulmonary failure, cerebrovascular lesion, pulmonary embolism, pneumonia, myocardial infarction, gastrointestinal hemorrhage, hepatic failure, intestinal ischemia, malignancy, aortic rupture/cardiac tamponade, and other. The categories used are similar to the ones proposed by Friberg et al. [13].

## Results

**Fig. 1:**
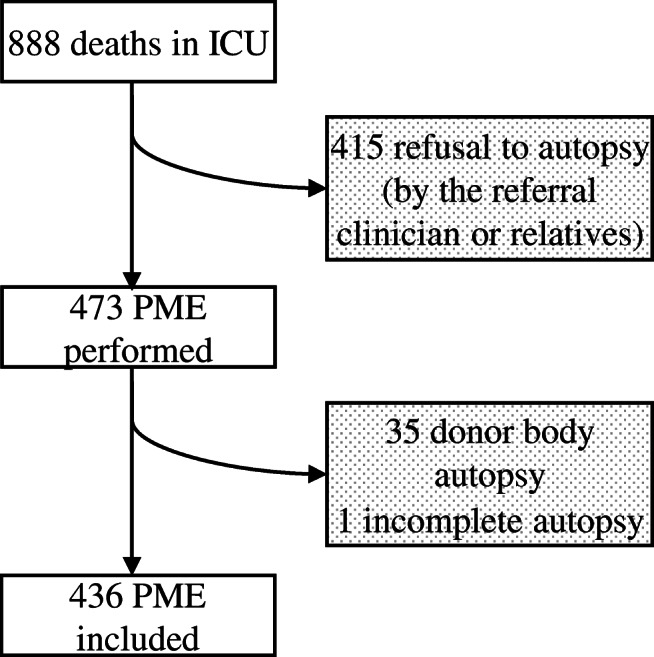
Flow chart summarizing the selection of the cases

During the 3-year period, 888 patients died in the ICU and 473 underwent PME, resulting in an ICU autopsy rate of 53.3%. Four hundred thirty-six PME were included in the present study (Fig. 1). The male to female ratio of the study group was 1.7 and the median age was 68 years old (varying from 22 to 97 years old). The median of hospitalization days was seven (1-112) of whom 3 days (1-75) were passed in the ICU. Thirty-four percent of patients (149) were admitted to the ICU after surgery of whom 31% (46) after cardiac surgery. The characteristics of the ICU patients who underwent PME are resumed in Table 2.

**Table 2:** Characteristics of the population included in the study (data for age, hospital stay, and ICU stay are presented as median)

*Characteristics of patients*	*2016*	*2017*	*2018*	*Total*
No. of patients	140	156	141	437
Age^a^ (years)	68 (25-94)	68.5 (2297)	66 (24-96)	68 (22-97)
Male/females	84/57	101/55	91/50	275/162
	59.3%/40.7%	64.7%/35.3%	64.5%/35.5%	62.9%/37.1%
Hospital stay^a^ (days)	6 (1-98)	6 (1-112)	8 (1-62)	7 (1-112)
ICU stay^a^ (days)	4 (1-74)	3 (1-75)	3 (1-33)	3 (1-75)
Patients admitted directly after surgery	48 (34.3%)	49 (31.4%)	52 (36.9%)	149 (34.1%)
Patients admitted directly after cardiac surgery^b^	17	18	11	46

^a^Results reported as: median (minimum-maximum)

^b^Cases also included in “Patients admitted directly after surgery” category

Autopsies revealed unexpected findings according to Goldman classification in 101 cases out of 437 (23.1%), which were classified as class I in ten autopsies (2.3%), class II in 34 (7.8%), class III in six (1.4%), and class IV in 51 autopsies (11.7%). Perfect agreement (class V) was observed in 76.9% of PME (336 autopsies). Forty-seven major discrepancies (class I and class II) were identified in 44 PME. Two cases presented two class I unexpected findings: invasive aspergillosis—pulmonary embolism and myocardial infarction—pulmonary embolism, respectively, and one case presented two class II unexpected findings: aspergillosis—tuberculosis.

The most frequent class I discrepancy identified was pulmonary embolism (3/12 class I findings) followed by invasive aspergillosis, myocardial infarction, and gastrointestinal perforation (one peptic ulcer perforation and one gastric perforation, both accompanied with sepsis and peritonitis), each one counting two cases. Other major discrepancies included gastric necrosis secondary to diaphragmatic hernia and one case of missed multi-metastatic carcinoma of unknown origin. The most frequent class II discrepancy were malignancies including metastatic and non-metastatic tumors (14/35), followed by pulmonary embolism (10/35). All causes of major discrepancies are listed in Table 3. The minor discrepancies (classes III and IV) included cardiac thrombi, hepatic cirrhosis, acute pancreatitis, pulmonary tuberculosis, and benign or malignant tumors not directly associated with the cause of death.

**Table 3:** Major discrepancies (Goldman class I and class II) found at PME

	*Causes*	*Number of discrepancies* (*n* = 47)
*Class I* (*n* = 12)	Pulmonary embolism	3
	Invasive Aspergillosis	2
	Myocardial infarction	2
	Gastrointestinal perforation	2
	Abdominal hematoma (not linked to previous surgery)	1
	Gastric necrosis	1
	Malignancy	1
*Class II* (*n* = 37)	Malignancy ^a^	13
	Pulmonary embolism	10
	Aspergillosis	4
	Gastro-intestinal perforation	2
	Myocardial infarction	1
	Retroperitoneal hematoma (not linked to previous surgery)	1
	Mesenteric infarction	1
	Pneumonia	1
	Tuberculosis	1
	Esophageal necrosis (unknown origin)	1

^a^Includes metastatic and non-metastatic neoplasms

Major discrepancies were found more frequently in patients hospitalized for less than 10 days then in the group with more than 10 days of hospitalization (13.8% vs 4.5%; *p* = 0.002). No statistical difference has been noticed concerning age, gender, and ICU stay. The cross-tabulated results are summarized in Table 4.

**Table 4:** Cross-tabulated results concerning the discrepancies and gender, age, length of stay in ICU, and duration of hospitalization

		*Class I-II*	*Class III-V*	*p value*
*Age*	< 60 years old	11 (8.2%)	123 (91.8%)	0.39
	≥ 60 years old	33 (10.9%)	270 (89.1%)	
*Gender*	Male	29 (10.5%)	246 (89.5%)	0.66
	Female	15 (9.3%)	147 (90.7%)	
*Hospital stay*	< 10 days	36 (13.8%)	224 (86.2%)	*0.002*
	≥ 10 days	8 (4.5%)	168 (99.5%)	
*ICU stay*	< 10 days	39 (11.4%)	302 (88.6%)	0.084
	≥ 10 days	5 (5.2%)	91 (94.8%)	

The cause of death was identified in 99.8% of cases (436/437). The major categories of cause of death identified in the autopsies over the 3-year period were cerebrovascular lesion (19%), sepsis/peritonitis (15.3%), and cardiopulmonary failure (14.2%), as described in Table 5. Divided by years, the order is maintained in 2016 and 2018, but in 2017 the main cause of death was myocardial infarction (16.7%) followed by cerebrovascular lesions (15.4%).

**Table 5:** Causes of death in autopsies

*Cause of death*	*2016*		*2017*		*2018*		*Total*	
	No.	%	No.	%	No.	%	No.	%
Cerebrovascular lesion	33	23.6	24	15.4	26	18.4	83	19.0
Sepsis/peritonitis	22	15.7	21	13.5	24	17.0	67	15.3
Cardiopulmonary failure	20	14.3	22	14.1	20	14.2	62	14.2
Myocardial infarction	14	10.0	26	16.7	13	9.2	53	12.1
Pneumonia	19	13.6	14	9.0	19	13.5	52	11.9
Hepatic failure	7	5.0	10	6.4	12	8.5	29	6.6
Malignancy	6	4.3	6	3.8	8	5.7	20	4.6
Pulmonary embolism	6	4.3	8	5.1	3	2.1	17	3.9
Gastrointestinal hemorrhage	1	0.7	7	4.5	5	3.5	13	3.0
Aortic rupture/cardiac tamponade	4	2.9	4	2.6	0	0.0	8	1.8
Intestinal ischemia	1	0.7	0	0.0	5	3.5	6	1.4
Not identified	0	0.0	1	0.6	0	0.0	1	0.2
Other	7	5.0	13	8.3	6	4.3	26	5.9

## Discussion

The present study reviewed the autopsies performed in ICU patients during a 3-year period. We report an autopsy rate of 53.3% which is slightly superior to the 41% mean reported (range 6-100%) in a systematic review performed by Winters et al. [14]. Shojania et al. [26] concluded that autopsy rates of 30-40% or higher are likely to produce fairly accurate estimates of the overall prevalence of major misdiagnosis in ICU patients, despite potential biases inherent in autopsy case selection which reinforce the accuracy of our study. Compared to the studies performed in the same institution in 2004 (autopsies performed in 1999) [24] and in 2007 [7] (autopsies performed in 2004-2005), we observed an increase of almost 10% and 20% respectively, underlying that despite the downward trend of autopsy practice worldwide over the years, we were able to maintain and even increase the number of ICU autopsies, emphasizing their central role in medical education, in evaluation of the accuracy of diagnostic imaging and in providing information on disease course and cause of death [8, 15, 21].

The total discrepancy rate observed was 23.1%, in line with the one described in 2004 (22.5%) and 2007 (21%) of whom 10.1% were related to the cause of death, with 2.3% being class I missed diagnosis and 7.8% class II. Our discrepancy rate is slightly inferior to the one reported by Winters et al. [14] with 28% of ICU autopsies reporting at least one misdiagnosis and similar to the 23.5% median error rate reported by Shojania et al. [16] in medical autopsies. The class I discrepancy rate is inferior to the 8% rate identified by Winters et al. [14] in a systematic review. Our results are consistent with previous results published by Podbregar et al. [27] or by Frohlich et al. [28] reporting both a 2.4% discrepancy rate in 170 autopsies of 373 deaths and 207 autopsies of 629 deaths, respectively. Other studies [29–35] reported class I discrepancy rates varying from 4 to 26%. This high discrepancy between studies can be explained by multiple factors: diagnostic error frequencies were higher with lower autopsy rates (usually in this situation only the most complex cases undergo autopsy [16, 31]), different ICU populations but also differences in the indications for autopsy. Even if the global discrepancy rate is higher than in our previous studies, the class I missed diagnosis found in 2.3% of cases are substantially below the 5.4% and 6% reported in 2004 and 2007, respectively, meaning a decrease of 57% and 62%. Previous studies [4, 16, 36–38] performed on ward, ICU, or unselected autopsies covering several decades showed a significant reduction of class I errors over the years, trend observed in our study too. Our results are inferior to the 6.3% projected prevalence of class I misdiagnosis for a hypothetical autopsy rate of 100% in ICU patients described by Winters et al. [14]. They concluded that “between 22600 and 40500 ICU patients die each year in the USA with and potentially from a diagnostic error and many more suffer a clinically relevant diagnostic error” underlining that despite medical and imaging progress, misdiagnosis still occurs and remains sufficiently high to encourage ongoing use of the autopsies. The most frequent class I error identified in our study was pulmonary embolism (three cases, 25%), followed by invasive aspergillosis, myocardial infarction, and gastrointestinal perforation with two cases each (17%). Podbregar et al. [27] and Tejerina et al. [32] reported also pulmonary embolism as the most frequent class I error. Other studies [29–31, 33, 34] point out myocardial infarction, infections as major missed diagnoses. Perkins et al. [31] concluded in 2003 that only 55% of patients undergoing PME had an electrocardiogram performed during the ICU stay, suggesting that the index of suspicion for ischemic heart disease is inappropriately low in the critically ill patients. A 10-year review focused on acute myocardial infarction diagnosed at autopsy concluded that “although acute myocardial infarction is an uncommon diagnosis rendered at hospital autopsy, a notable subset of cases demonstrates diagnosis discrepancy between the clinical impression and ultimate pathologic diagnosis. Interestingly, most cases in the series are not related to plaque disruption and thus best classified as a type two myocardial infarction, which is associated with imbalance between oxygen demand and supply” [39]. Moreover, diagnosing acute myocardial infarction in PME depends on the time lapse between the onset and the death. As underlined by Kurata [40] and Sabatasso et al. [41], earliest findings of acute myocardial infarction including contraction band necrosis may be subtle or nonspecific and it takes at least 1 h after the onset of the attack while evident neutrophilic infiltration occurs 6 to 12 h after the onset of the ischemic attack.

One study [35] identified hemorrhage as the most frequent class I error. Compared to Maris et al. [7], we improved the diagnostic accuracy for detecting myocardial infarction (from 8/55-14.5% in 2007 to 3/48-6% in 2020) and aspergillosis (7/17 errors, around 40%). Despite the improvement, it remains important to recognize typical and atypical presentations of infection for fast and effective therapy, especially in immunocompromised patients. More than three decades ago, Goldman et al. [8] reported similar unexpected findings in a non-ICU autopsy population, including pulmonary embolism, acute myocardial infarction, tumors, and infections. Despite advanced medical techniques, these diagnoses remain difficult to identify, being also nowadays frequently reported as main clinical missed diagnoses. Therefore, we emphasize the importance of maintaining a high index of suspicion for these diagnoses in critically ill patients consistent with previous recommendations [7, 33].

The incidence of class II errors is in line with previous reports (3.1-26.3%) [27, 30–35]. We observed more class II misdiagnoses compared to the study performed in our institution in 2004 but less compared to 2007 (3.1% and 13%, respectively). The most frequent class II error was malignancy (metastatic and non-metastatic) representing almost 40% (13/35) of errors, in slight increase compared to 2007 (31%, 12/38). In spite of an increased use of diagnostic procedures such as computed tomography, undiagnosed neoplasms remain among major missed diagnoses [30, 31]. The high discordance rate can be explained by the fact that malignancies may be masked by more acute problems in critically ill patients. Usually, malignancies represent important comorbidities that may influence the patient’s management, but they do not represent often the main cause of death. The study performed in the same institution by Dimopoulos et al. [24] identified disseminated aspergillosis and pulmonary embolism as the most frequent major discrepancies (class I/II) summing up 31% (6/19) and 26% (5/19) but no misdiagnosed malignancy was identified. Extensive results of other studies on autopsies performed in ICU patients are presented in *Electronic Online Resource* 1*.*

We identified an important relationship between the length of hospital stay and the type of discrepancy detected. We found that in patients hospitalized fewer than 10 days, the detected discrepancies were mainly major (class I and II), while in patients with longer stay, minor discrepancies that are not directly related to the cause of death were more frequent. We suggest that a longer stay allows more detailed investigations and consequently a decrease in the misdiagnosis frequency. Regarding the ICU stay, we detected more major discrepancies in patients staying less than 10 days, but the relationship was not statistically significant. The study performed on the “99 autopsy series [25] showed a statistically significant relationship between the length of ICU stay and the type of discrepancy detected, concluding that following a short ICU stay, PME can detect findings whose diagnosis is difficult even though they may be suspected by the intensivist” while on the “04-05 autopsy series” [7], a statistically significant higher rate of major discrepancies in patients staying more than 10 days in the ICU. Data reporting an association between the length of ICU/hospital stay and frequency of major discrepancies is very heterogeneous. Mort and Yeston [9] reported two decades ago that patients staying longer than 48 h in ICU department were more likely to have a major discrepancy than those who died within 48 h. Other studies reported that the length of ICU or hospital stay did not influence the frequency of major discrepancy [30, 33, 35]. In several studies, the number of missed diagnoses increased with the age of patients [42, 43] based on the consideration that elderly patients usually present multiple comorbidities and an unclear clinical presentation [33]. Dimopoulos et al. [24] showed that minor discrepancies were more common in older patients (> 50 years old) than in younger patients, but no statistically significant relationship between age and number of missed discrepancies. However, this fact was not observed in our PME series, corroborating previous investigations in which age of patients was not significantly different between those with or without missed diagnoses [7, 33, 44]. We observed a predominance of PME in male patients, but there is no relationship between gender and the incidence of missed diagnosis, results that can be corroborated with both studies previously performed in our institution.

A thorough analysis of main death causes revealed significant disparities between studies that may be explained by several aspects. Firstly, the healthcare access and quality are unequal worldwide [45] translating to different disease patterns between industrialized and developing countries, and, thus, different leading causes of death. The leading causes in high-income countries are related mainly to cardiovascular diseases, while in low-income countries, infectious pathology is responsible for many deaths. For instance, a Swedish study [13] reported cardiovascular events (including cardiopulmonary failure, myocardial infarct, pulmonary embolism, aortic rupture/cardiac tamponade, cerebrovascular lesion, intestinal ischemia) responsible for the majority of deaths (71.4%), while infections (including pneumonia, sepsis/peritonitis) were identified in 15.8% of cases. Conversely, a study performed in Mozambique [46] determined infections (viral, bacterial, fungal, and parasitic) as the main death cause, counting 70.4%, while cardiovascular events were responsible for 9% of cases. Considering demographic, socio-economic factors, and access to healthcare, the Belgian population is similar to the Swedish one. We equally report cardiovascular disease as the main cause of death (52.4%), but a higher prevalence of infections (27.2%). Secondly, the comparison of death causes among different population is limited by different indications for PME between countries and, furthermore, between ICU and ward patients. For instance, our protocol implies systematic PME on the ICU patients except when opposition of the family. In the non-ICU units, autopsy is requested only when unexpected death occurs and only after family consent. Moreover, our hospital is recognized as a tertiary referral center for cerebrovascular disease, leading to a specific population recruitment in the ICU and, thus, for PME, due to a high mortality rate [47]. We report significantly more deaths caused by cerebrovascular disease than reported by Friberg et al. [13] (19% versus 5%).

We acknowledge the limitations of this study. First, this is a retrospective study and the diagnostic work-up of each individual case was not critically reviewed. Second, there is no standard procedure for the selection of patients for autopsy. In the present study, almost half of the patients who died in the ICU underwent PME, representing another potential limitation, although our autopsy rate is higher than in many other studies. Third, a number of patients died after therapeutic limitations as a result of withholding or withdrawing life support decisions that were not reported in the present study. The major strength of this study is the large number of patients hospitalized in a large medico-surgical department of intensive care and the important number of performed autopsies. Nevertheless, these findings from an academic hospital may not be applicable to other ICUs. This study may serve as the basis for further research focused on understanding the persistence of discrepancies despite the advanced medical techniques.

## Conclusion

Our study showed that the discrepancies between clinical and PME diagnoses persisted in spite of the progress in medical skills and the use of highly sensitive and specific tests. The comparison between clinical and postmortem diagnoses is critical and should be seen as a useful tool to improve patient care in an attempt to reduce the misdiagnoses. Beside this, PME remains a powerful tool in medical education and a fundamental element of quality control in medicine. Secondly, the autopsy after a short hospital stay may reveal unexpected findings whose diagnosis is challenging even if it may be suspected by the intensivist.

## Supplementary information

ESM 1 (DOCX 31 kb).

## Data Availability

The datasets analyzed during the current study are available from the corresponding author on reasonable request.
