# Supplementary material for: Comparison of antemortem clinical diagnosis and post-mortem findings in intensive care unit patients
Source: Virchows Arch. 2021 Feb 13;479(2):385–92. doi: 10.1007/s00428-020-03016-y (PMC8364530; doi:10.1007/s00428-020-03016-y)
Supplement: Supplementary file 1 — (DOCX 31 kb). [file 428_2020_3016_MOESM1_ESM.docx]

*Comparison of antemortem clinical diagnosis and post-mortem findings in intensive care unit patients*

*Virchows Archiv*

Stefan Rusu, Philomene Lavis, Vilma Salgado, Marie-Paule Van Craynest, Jacques Creteur, Isabelle Salmon, Alexandre Brasseur, Myriam Remmelink

*The affiliations of the corresponding author:*

1. Hôpital Erasme, Université Libre de Bruxelles, Department of Pathology, Brussels, Belgium
2. Centre Universitaire Inter Regional d'Expertise en Anatomie Pathologique Hospitalière (CurePath), B-6040 Charleroi (Jumet), Belgium

*E-mail address of the corresponding author*: [myriam.remmelink@erasme.ulb.ac.be](mailto:myriam.remmelink@erasme.ulb.ac.be)

*Electronic supplementary material 1: Autopsy studies conducted in adult ICUs and the results reported*

| Study (year) | Country | Type of Study | Length of study (months) | No of autopsies/ total number of deaths | Autopsy rate | Population | Discrepancy rates | Class I findings | Class II findings | Main missed diagnosis | Comments |
| --- | --- | --- | --- | --- | --- | --- | --- | --- | --- | --- | --- |
| Maris et al. [1](2007) | Belgium | Retrospective | 24 | 289/786 | 37% | Mixed ICU | 21% | 6% | 13% | Class I- aspergillosis (7/17), pulmonary embolism (3/17); Class II-malignancies (12/38), myocardial infarction (6/38) | Major discrepancies more frequently in patients with >10 days in ICU than those < 10 days (p=0.008) |
| Dimopoulos et al[2] (2004) | Belgium | Retrospective | 12 | 222/489 | 45.4% | Mixed ICU | 22.5% | 5.4% | 3.1% | Malignancy, fungal infections | Fewer discrepancies in patients staying < 10 days than in those staying >10 days (P<0.001)  Minor discrepancies more common in older (>50 y.o.) than in younger patients (<50 y.o.) (p<0.05) |
| Podbregar et al [3] (2001) | Slovenia | Retrospective | 24 | 126/270 | 46.6% | Medical ICU | 52% | 9.5% | Not specified | 7/12 due to unrecognized septic origin (pyonephrosis, pyometritis, perinephric abscess, …) | No level of clinical diagnostic certainty could predict the pathological findings  Autopsies are performed more often in younger patients without chronic disease and in patients with a low clinical diagnostic certainty |
| Podbregar et al [4] (2011) | Slovenia | Retrospective | 20 | 170/373 | 45.6% | Medical ICU | 11.8% (only major discrepancies considered) | 2.4% | 9.4% | Class I- massive pulmonary embolism (75%) | Problematic interpretation of bedside diagnostics by inexperienced physicians in class I major discrepancies |
| Combes et al ^4^ (2004) | France | Prospective | 36 | 167/315 | 53% | Mixed ICU | 68.9% | 10.2% | 26.3% | Malignancy (12.3%), myocardial infarction (6.4%), stroke (7%) | Correct diagnosis would have changed management and possibly resulted in a cure or prolonged survival for up to 10% of the patients.  41% of the patients with class I discrepancies were immunocompromised on ICU admission |
| Perkins et al [5] (2003) | Great Britain | Retrospective | 42 | 49/636 | 7.7% | Mixed ICU | 55% | 26% | 13% | Class I- myocardial infarction (3/10)  Class II- malignancy (4/5) | No relationship between the incidence of major missed diagnoses and age, Acute Physiology and Chronic Health Evaluation II score, or duration of ICU stay |
| Tejerina et al[6] (2012) | Spain | Prospective | 300 | 866/2857 | 30.3% | Mixed ICU | 18.5% (only Class I and II) | 7.5 | 11 | Pulmonary embolism (15%), pneumonia (14.5%), secondary peritonitis (8%) | Rate of diagnostic discrepancy remained relatively constant over time, and the conditions leading to discrepancies have slightly changed, with pneumonia showing a decline in diagnostic accuracy in the last years. |
| Tejerina et al [7] (2018) | Spain | Prospective | 96 | 215/671 | 32% | Mixed ICU | 17.7% (only major discrepancies) | 8.4% | 9.3% | Invasive aspergillosis (7/38), intestinal ischemia (4/38), myocardial infarction (4/38), malignancy (3/38) | No statistically significant correlation between any premortem factor, including age, sex, severity of illness, length of hospital stay before ICU admission, length of ICU stay before death, duration of mechanical ventilation, or admitting unit, and the level of agreement between clinical and pathological diagnosis |
| Nadrous et al[8] (2003) | USA | Retrospective | 36 | 527/1597 | 33% | Mixed ICU | 21% (only class I and II) | 4 | 17 | Class I- cardiac tamponade (4/11), fungal infection (3/11), bacterial infection (3/11)  Class II-infections (19/78), cancers (12/78) | No significant differences in age, sex, or length of stay in the ICU or hospital among patients with and without diagnostic errors or among patients with type I and II errors. |
| Twig et al[9] (2001) | Great Britain | Retrospective | 36 | 97/252 | 40.5% | Mixed ICU | 23.7 | 4.1 | 19.6 | Discrepancies fell into four main groups; unrecognized hemorrhage (7 patients), myocardial infarction (5), thromboembolic disease (5) and infectious complications (4). |  |
| Frohlich et al [10] (2014) | Ireland | Retrospective | 60 | 207/629 | 32% | Mixed ICU | 21% | 2.4 | 5.4 | Class I- pancreatic abscess, pneumocystis’ pneumonia, mesenteric ischemia, aortic dissection  Class II- myocardial infarction (4/11) | Lower rate of clinicopathological discrepancy in critically ill patients than previously reported. |
| Present study | Belgium | Retrospective | 36 | 473/888 | 53.3% | Mixed ICU | 23.1% | 2.3 | 7.8 | Class I-pulmonary embolism (3/12)  Class II- malignancy (13/35) | Major discrepancies more frequent in patients hospitalized for less than ten days then in patients with more than ten days of hospitalization (p=0.002); no statistical difference noticed concerning age, gender and ICU stay. |

*Bibliography*

1. Maris C, Martin B, Creteur J, et al (2007) Comparison of clinical and post-mortem findings in intensive care unit patients. Virchows Arch Int J Pathol 450:329–333. https://doi.org/10.1007/s00428-006-0364-5

2. Dimopoulos G, Piagnerelli M, Berré J, et al (2004) Post mortem examination in the intensive care unit: still useful? Intensive Care Med 30:2080–2085. https://doi.org/10.1007/s00134-004-2448-5

3. Podbregar M, Voga G, Krivec B, et al (2001) Should we confirm our clinical diagnostic certainty by autopsies? Intensive Care Med 27:1750–1755. https://doi.org/10.1007/s00134-001-1129-x

4. Podbregar M, Kralj E, Čičak R, Pavlinjek A (2011) A triad algorithm for analysing individual ante- and post-mortem findings to improve the quality of intensive care. Anaesth Intensive Care 39:1086–1092. https://doi.org/10.1177/0310057X1103900617

5. Perkins GD, McAuley DF, Davies S, Gao F (2003) Discrepancies between clinical and postmortem diagnoses in critically ill patients: an observational study. Crit Care 7:R129–R132

6. Tejerina E, Esteban A, Fernández-Segoviano P, et al (2012) Clinical diagnoses and autopsy findings: discrepancies in critically ill patients*. Crit Care Med 40:842–846. https://doi.org/10.1097/CCM.0b013e318236f64f

7. Tejerina EE, Padilla R, Abril E, et al (2018) Autopsy-detected diagnostic errors over time in the intensive care unit. Hum Pathol 76:85–90. https://doi.org/10.1016/j.humpath.2018.02.025

8. Nadrous HF, Afessa B, Pfeifer EA, Peters SG (2003) The role of autopsy in the intensive care unit. Mayo Clin Proc 78:947–950. https://doi.org/10.4065/78.8.947

9. Twigg SJ, McCrirrick A, Sanderson PM (2001) A comparison of post mortem findings with post hoc estimated clinical diagnoses of patients who die in a United Kingdom intensive care unit. Intensive Care Med 27:706–710. https://doi.org/10.1007/s001340100903

10. Fröhlich S, Ryan O, Murphy N, et al (2014) Are autopsy findings still relevant to the management of critically ill patients in the modern era? Crit Care Med 42:336–343. https://doi.org/10.1097/CCM.0b013e3182a275b1
